# The role of B lymphocytes in the immuno-biology of non-small-cell lung cancer

**DOI:** 10.1007/s00262-019-02461-2

**Published:** 2020-01-04

**Authors:** Akshay J. Patel, Alex Richter, Mark T. Drayson, Gary W. Middleton

**Affiliations:** 1 Institute of Immunology and Immunotherapy (III), College of Medical and Dental Sciences, University of Birmingham, Vincent Drive, Birmingham, B15 2TT UK; 2 School of Cancer Sciences, University of Birmingham, Birmingham, UK

**Keywords:** B lymphocyte, Non-small-cell lung cancer (NSCLC), Humoral Immunity, Immunoglobulin, Tumour -infiltrating B lymphocyte (TIL-B)

## Abstract

Tumour-infiltrating immune cells have been widely implicated to play a significant role in carcinogenesis, through both pro- or anti-tumour effects. The multi-faceted effects of lung cancer associated T lymphocytes have been extensively studied, and yet, the role of B lymphocytes remains an area less studied. In this review, we will describe the current understanding of the role of tumour-infiltrating B lymphocytes in NSCLC, discuss their prognostic significance, their functionality within the tumour microenvironment and ultimately how we might harness B-cell biology to develop B-cell therapeutic strategies in cancer.

## Introduction

Immune responses within the tumour microenvironment (TME) are important determinants of tumour behaviour, progression, and aggressiveness [1]. The role of T-cell-mediated immune responses in solid tumours is well established and has become the driving force for major therapeutic advances, specifically with the advent of immune checkpoint inhibitors [2]. The success of single agent and combination checkpoint blockade, which enables antigen-experienced microenvironmental T cells, has transformed the treatment paradigm of advanced NSCLC both as a front-line option and in the platinum-refractory setting. As a consequence, our understanding of the biology of these T cells has expanded exponentially. In contrast, our knowledge of B-cell biology in cancer is less well developed. Tumour-infiltrating B cells have been observed in all stages of lung cancer development, with their presence differing according to stage and histological subtype. Given that they play a role in both humoral and cellular immunity B-cell parameters may be important in determining both responsiveness to and toxicity of checkpoint blockade. Manipulation of B-cell biology might offer significant immune-therapeutic opportunities [3]. Thus, understanding B-cell biology in NSCLC is of fundamental importance in informing potential novel multi-faceted approaches to favourably reset the immune contexture of the cancer microenvironment.

## B-cell ontogeny

In the bone marrow, haematopoietic stem cells serve as the common lymphoid progenitor and continually give rise to B cells throughout life [4]. B-cell development and differentiation are subsequently regulated through the B-cell receptor (BCR). On leaving the bone marrow, B-cell development takes place in B-cell follicles within secondary lymphoid organs (SLOS), where germinal centres (GC) develop in response to antigen encounter. Mature B cells undergo a process known as somatic hypermutation (SHM), where enzyme induced mutations are introduced in the heavy and light chain variable regions to further diversify the immunological repertoire. The same enzyme mediates immunological class switch recombination (CSR), replacing the μ constant region for another heavy chain region to generate IgA, IgE or IgG. The cells with the highest affinity B-cell receptors (BCR) are positively selected (this requires signalling through the BCR for the cell to survive, negative selection occurs when the B-cell antigen receptor binds self-antigen and, therefore, undergoes cell death) and undergo clonal expansion in the germinal centre. These class-switched B cells can then give rise to long-lived plasma cells or memory B cells [5]. Germinal centres, which are normally found in SLOS such as the spleen and lymph nodes, can also occur as tertiary lymphoid structures (TLS) in tumours; this has been reported in colon, breast and NSCLC [6–8]. The de novo formation of ectopic lymphoid tissue can occur at the site of inflammation in potentially any organ system [9]. There is evidence that the adaptive immune system can be initiated independent of SLOS in these TLS in NSCLC [7].

## B-cell function

### Antigen presentation

T cell immune responses can be activated by B cells. Antigen-specific interactions require antigen internalisation via the BCR with subsequent presentation to T cells in an MHC-restricted manner [10]. Depleting host B cells from normal adult mice results in sub-optimal CD4 + T cell activation during immune responses to low-dose foreign antigen [11]. Activated B cells derived from peripheral donor blood from healthy controls present antigens to CD4 + and CD8 + T cells [12]. B cells and dendritic cells further provide a co-stimulatory signal which is critical to the expansion of an effector T cell population, namely the cross-linking of CD40 with CD154 (CD40 Ligand) on CD4 + T cells [10] which in turn induces the expression and thence stabilisation of CD86 (B7.2) and CD80 (B7.1) on the B-cell surface (second co-stimulatory signal).

### Antibody production

B cells exist as long-lived plasma cells to produce antigen-specific antibody. The functional BCR can recognise an array of foreign antigens in the circulation and lymphoid organs, which then triggers antigen-specific antibody responses. Following antigen binding, BCR stimulation results in a signalling cascade mediated by membrane bound protein tyrosine kinases, Spleen Tyrosine Kinase (SYK), Bruton Tyrosine Kinase (BTK) and PI3K, in particular PI3Kδ which is a p110 isoform with high lymphocyte specificity [4]. This in turn results in B-cell differentiation into plasma and memory cells. The plasma cells will traffic to the bone marrow or reside in SLOS, where they will continue to secrete antibody. BCR signalling controls this process from early B-cell precursor development to terminal plasma cell differentiation, long-lived IgG plasma cells are devoid of the BCR [5].

### Immunosuppression

B cells play a vital role in the development of the immune system but are also responsible for immune homeostasis. Their immunosuppressive role has been illustrated by their ability to function as regulatory cells or Bregs, whereby they influence T cell differentiation and thus T-cell-mediated inflammatory responses through IL-10 production [13]. B regulatory cells are associated with limiting excessive inflammation [14] and mice lacking IL-10 producing Bregs develop chronic inflammation [15]. IL-10 producing Bregs induce the Treg phenotype by skewing T cell differentiation in mice [16] and humans [17]. Other cytokines that have been implicated in B-cell specific immunosuppression are TGF-beta and IL-35. TGF-beta when produced by B cells can induce CD4 + T cell apoptosis [18] and CD8 + T cell anergy [19]. Chimaeric mice lacking either p35 or EBi3 (IL-35 subunits) in B cells develop accentuated autoimmune responses and have greater protection against Salmonella induced sepsis [20].

IL-10 has been useful for Breg characterisation in both mice and humans; however, this can be up or down-regulated during immune activation and is not a stable inducible trait [14, 21]. A lineage-specific marker for Bregs equivalent to Foxp3 has not been identified. B cells are polarised to Bregs in response to microenvironmental cues: Bregs have been derived by treating human derived peripheral B cells with tumour-conditioned media; these Bregs were able to suppress the activity of human T cells in vitro and they exhibited low surface expression of CD20 unlike healthy control human B cells [22, 23]. These tumours evoked Bregs did not utilise IL-10 dependent suppression but instead primarily functioned to promote the differentiation of Tregs (CD25+) via the TGFβ signalling axis [22].

## Pre-clinical studies of the role of B cells in cancer

There appears to be a significant difference in the role of B cells in animal models which represent different stages in the development of cancer. In murine models of pre-malignancy, B cells appear to drive inflammation which in turn induces pre-malignancy.

### Pre-malignancy models

Early evidence from the K14-HPV16 model (these RAG-1 knockout (KO) mice in which carcinogenesis is initiated by HPV lack T and B cells) has shown that the lack of an adaptive immune response results in failure to initiate leucocyte infiltration during pre-malignancy [24] and this halts progression towards carcinogenesis. Adoptive transfer of B lymphocytes or serum from HPV16 mice into HPV16/RAG1−/− mice resulted in significant infiltration of CD45 + leucocytes, macrophages and granulocytes in the dermal stroma as well as detectably higher levels of serum Ig which then enhanced malignant progression. In this model of squamous carcinogenesis, B cells were shown to play a role in activating Fcγ receptors (FcγRs) on resident and recruited myeloid cells likely via circulating immune complexes (CIC) detected in the serum of HPV16 mice and formed from IgG bound to cognate HPV16 antigens. These CICs were localised to both the epidermal and dermal components of neoplastic skin. The recruitment of these chronically activated leucocytes was dependent on FcγR expression [25], as shown by the reduced level of inflammatory cell infiltration in neoplastic skin in FcγR−/− KO mice. Subsequently it was shown in K14-HPV16 mice that premalignant dysplasia could be prevented through B-cell depletion [26]. Administration of anti-CD20 monoclonal antibodies depleted B cells in peripheral blood and SLOS with resultant reduced levels of circulating IgG, immune complex deposition and trafficking of myeloid cells (CD11 +), CD45 + leucocytes, mast cells and GR1 + cells to neoplastic skin [26].

In a DMBA/TPA murine model of skin carcinogenesis adoptive transfer of B cells from DMBA/TPA wild type (WT) mice into TNF-α knockout mice significantly increased papilloma development (*p* < 0.05), an effect not seen when B cells from the TNF-α knockout mice were transferred to RAG2−/− mice [27]. TNF-α is a potent inflammatory cytokine and tumour promotor, Bregs are known producers of TNF-α. Selective TNF-α deletion in CD19 + -Cre B cells significantly reduced papilloma development compared to B cells from WT mice (*p* < 0.002). TNF-α knockout mice showed increased levels of IFN-γ and CD8 + T cell skin infiltration, but also a significant reduction in the number of splenic CD19 + CD21hi, IL-10 producing Bregs compared with WT mice (*p* < 0.01). Further experimental data showed that TNF-α blockade of LPS-induced B-cell activation significantly reduced IL-10 production with no difference in IL-2, -4, -5, -12 or IFN-γ. The results of this study identify Bregs as contributory to squamous carcinogenesis, with their activity likely regulated by TNF-alpha with Bregs themselves acting as a cellular source of TNF-α.

The impact of B cells in established cancer appears to be very different to their role in pre-malignant disease.

### Established cancer models

B-cell depletion using anti-CD20 in a B16 melanoma murine model resulted in increased tumour burden and the development of pulmonary metastasis [28]. CpG (TLR 9 ligand) primed B cells caused tumour regressions in a B-cell deficient melanoma model [29]. CpG treatment of B cells induced higher expression of MHC class I and II as well as CD20. It was also noted that in the mice that underwent adoptive B-cell transfer, the lung tumours expressed significantly lower levels of BCL-2 (an anti-apoptotic protein) and increased levels of TRAIL (TNF-related-apoptosis-inducing-ligand, a pro-apoptotic protein), TRAIL expression is highest in germinal centre B cells and thus the upregulation in these murine lung tumours is likely B-cell driven [29].

In a 4T1 breast cancer model treatment with an anti-CD20 antibody resulted in massive cancer growth and metastasis. Eradication of CD20^hi^B cells, enriched for a select population of CD20^LO^ Bregs which escaped CD20-directed depletion and thus significantly suppressed CD4 + and CD8 + T cell activity thus abrogating anti-tumour responses [23]. Targeted delivery of CpG-ODN to CXCR5 expressing cells, reversed the phenotype of these tumour evoked Bregs (which upregulated CD20) and restored effector B-cell responses. This was demonstrated by a complete abrogation of tumour metastasis in anti-CD20 treated 4T1.2 cancer bearing mice after adoptive transfer of CpG treated B cells [30]. Using the same murine model, Tao et al. demonstrated that IL-10 inhibition significantly augmented the therapeutic efficacy of adoptive B-cell transfer, as demonstrated by increased trafficking of CD8 + T cells into the tumour microenvironment as well as in vitro antigen-specific B-cell dependent, FasL-mediated tumour cell killing [31]. Activated B cells from this model produce IgG and mediate complement-dependent tumour cell lysis in vitro [32]. Finally, the use of intra-tumoural TLR9 immune stimulation in combination with PD-1 blockade has demonstrated clinical efficacy in advanced melanoma, with increased B-cell infiltration noted on post-treatment tumour biopsies [33]. Phase I clinical trial data from patients with metastatic solid tumours showed a non-significant increase in TLR9-expressing naïve B cells during therapy [34].

## Tumour infiltrating B lymphocytes in human lung cancer

Table 1 summarises studies that have identified TIL-Bs in tissue and examined their associated prognostic significance. These studies largely focus on NSCLC which is where the correlation between TIL-Bs and disease-specific outcome has been shown to be strongest by comparison with other forms of Lung cancer. However, groups have investigated the immune milieu in large cell carcinoma and Small Cell Lung Carcinoma (SCLC). In the former, Eerola et al. have demonstrated that TIL-Bs do correlate with better overall survival [35], whereas in SCLC, B-cell infiltration is significantly reduced compared with CD8 + T cell and Macrophage infiltration and TIL-B number has not been shown to associate with survival (*p* = 0.634 by log rank) [36].

**Table 1:** Prognostic significance of TIL-Bs in

Study	Markers	Tumour classification	Methods	No. of cases	Follow-up (months)	Outcome and prognostic significance
Pelletier et al. [47]	CD20	Stage I–IV, Adenocarcinoma, Squamous Cell Carcinoma, Adeno-squamous and Large Cell Carcinoma	IHC	113	120	CD20 + B cell presence in the peri-tumoural region was positively prognostic (*p* = 0.04); this association was even stronger in non-squamous cell carcinomas (*p* < 0.001)
Al-Shibli et al. [46]	CD20	Stage I–IIIa, Adenocarcinoma, Squamous Cell Carcinoma, Large Cell Carcinoma and Broncho-alveolar Carcinoma	IHC	335	60	Epithelial and stromal CD20 + B cell presence (*p* = 0.023 and *p* < 0.001, respectively) correlated with improved disease-specific survival
Kinoshita et al. [45]	CD20	Stage I or III, Adenocarcinoma or non-adenocarcinomas	IHC	218	120	Multivariate analyses identified low accumulation of CD20 + B cells as an independent worse prognostic risk factor in adenocarcinomas (HR; 1.71, *p* = 0.004 for recurrence free survival). A high accumulation of CD20 + B cells was a favourable prognostic factor in non-smokers with adenocarcinoma (hazard ratio; 0.22, *p* = 0.03 for overall survival, *p* = 0.009 for recurrence free survival)
Banat et al. [51]	CD20	Stage I–III, Adenocarcinoma, Squamous Cell Carcinoma, Adeno-squamous and Small Cell Carcinoma	IHC	70	ND	Stage dependent changes were documented, whereby CD20 + B cell number was elevated in NSCLC tissue and higher in stage III tumours relative to stage I tumours, this was independent of tumour size and type. This group also showed a considerably higher number of CD20 + cells in N0 tumours compared with N1 + 2 samples. A higher density of MUM-1 positive plasma cells was found in NSCLC tissue compared with healthy control lungs. This finding bore no correlation with stage, size, histology or nodal status
Hernandez-Prieto et al. [42]	CD20 CD79	Stage I or II, Adenocarcinoma, Squamous Cell Carcinoma, Adeno-squamous and Large Cell Carcinoma	IHC	84	24	A weak presence of CD20 + B cells was found in the tumour microenvironment of the high-risk relapse subgroup of stage I/II NSCLC
Eerola et al. [35]	CD20	Stage I–III, Large Cell Carcinoma	IHC	38	60	Higher intra-tumoural CD20 + B cell infiltration correlated with significantly higher survival than those with low numbers of B cells; *p* = 0.05
Schalper et al. [48]	CD20	Stage I–IV, Adenocarcinoma, Squamous Cell Carcinoma	IF	202 (YTMA79)	60	Higher CD20 + B cell number associated with significantly longer overall survival, HR = 0.523, *p* = 0.004
Schalper et al. [48]	CD20	Stage I–IV, Adenocarcinoma, Squamous Cell Carcinoma	IF	350 (YTMA140)	60	Increased CD20 + B cell number was not associated with improved NSCLC overall survival, HR = 0.887, *p* = 0.447
Hald et al. [52]	CD20	Stage I–IIIa, Adenocarcinoma, Sqaumous Cell Carcinoma, Large Cell Carcinoma	IHC	371	60	Epithelial and Stromal CD20 expression was not deemed a prognostic factor for survival in NSCLC, *p* = 0.059
Suzuki et al. [51]	CD20	Stage Ia or Ib Adenocarcinoma	IHC	455	60	Tumoural and Stromal CD20 + B cell expression had no significant prognostic value for patients with stage I Adenocarcinoma, *p* = 0.417 and *p* = 0.389, respectively
Kurebayashi et al. [54]	CD20	Stage I–IIIa, Adenocarcinoma only	IHC	111	85	The infiltration of inter-follicular B cells in cancer stroma was significantly associated with a poorer prognosis when analysed for all adenocarcinoma cases and stage I cases alone; *p* < 0.001 for stage I and all adenocarcinoma cases
Germain et al. [44]	CD20	Stage I to II, Adenocarcinoma, Squamous Cell Carcinoma	IHC	74	60	The size and density of B cell follicles along with the size and density of germinal centres and the number of antibody-producing plasma cells in tumour-associated TLS positively correlated with better clinical outcome in NSCLC in both early and advanced stage; for overall survival, HR 11, *p* = 0.02
Gottlin et al. [8]	BCL-6, CD21-Germinal Centre B cell Markers	Stage I–IV, Adenocarcinoma, Squamous Cell Carcinoma, Large Cell Carcinoma and Broncho-alveolar Carcinoma	IHC	91	34	NSCLC specimens have displayed evidence of immune response to antigen stimulation; germinal centres were seen within the tumour and at the tumour margin; moreover, early stage I NSCLC tumours displayed a higher prevalence of intra-tumoral germinal centre formation compared with higher stage tumours (II–IV); *p* < 0.02
Al-Shibli et al. [46]	CD138	Stage I–IIIa, Adenocarcinoma, Squamous Cell Carcinoma, Large Cell Carcinoma	IHC	191	192	Epithelial and Stromal CD138 + cell infiltration showed no significant correlation with Disease-free survival, *p* = 0.847.
Hald et al. [52]	CD138	Stage I–IIIa, Adenocarcinoma, Sqaumous Cell Carcinoma, Large Cell Carcinoma.	IHC	371	60	Epithelial and Stromal CD138 + cell infiltration did not correlate with survival in NSCLC, *p* = 0.292
Kurebayashiet al. [54]	CD138 p63	Stage I–IIIa, Adenocarcinoma only	IHC	111	85	The infiltration of inter-follicular/para-follicular plasma cells in cancer stroma was significantly associated with poorer prognosis when analysed for all cases of lung ADC and for stage I cases alone (*p* < 0.001). The plasma cell infiltration is the independent negative prognostic factor in Grade1/2 papillary/acinar ADC (*p* = 0.006)
Lohr et al. [56]	CD138	Stage I–IV, Adenocarcinoma, Squamous Cell Carcinoma, Large Cell Carcinoma	IHC	355	58.7	CD138 expression associated with increased survival across all cases of NSCLC (HR = 0.74, *p* = 0.041) but particularly for adenocarcinoma (HR = 0.54, *p* = 0.004)
Lohr et al. [56]	Ig-kappa-C	Stage I–IV, Adenocarcinoma, Squamous Cell Carcinoma, Large Cell Carcinoma	IHC	355	58.7	Immunoglobulin kappa C (IGKC) expression was independently associated with longer survival (HR = 0.72, *p* = 0.035) with a stronger pre-dilection for the adenocarcinoma subtype (HR = 0.57, *p* = 0.013), for recurrence-free survival, *p* = 0.044. IGKC expression correlated strongly with CD138 expression (*p* < 0.0001)
Fujimoto et al. [55]	IgG-4	Stage I–IV, Adenocarcinoma, Squamous Cell Carcinoma, Large Cell Carcinoma, Adeno-squamous and Sarcomatoid Carcinoma	IHC	294	60	In stage I NSCLC, IgG4 + plasma cell stromal infiltration correlated with favourable prognosis (*p* = 0.04); overall survival, *p* = 0.0409 for SCC. *p* < 0.0001 for Adenocarcinoma
Schmidt et al. [43]	Ig-kappa-C	Stage I–IV, Adenocarcinoma, Squamous Cell Carcinoma	qRT-PCR Microarray	196	120	Gene-expression profiles were analysed in various types of human solid cancers, IGKC expression consistently associated with longer survival in NSCLC on uni- and multivariate analyses (*p* < 0.001 and *p* = 0.032, respectively); moreover, the source of IGKC expression was exclusively the tumour-infiltrating plasma cell. For Adenocarcinoma, overall survival, p = 0.002.
Zhang et al. [68]	CD19+ CD5+ Stat3+	Lung and Prostate Cancer	IF IHC	9	ND	In lung tumour lymph nodes, p-STAT3 expression and CD5 positivity in CD19 + B cells correlated. However, the limited number of patient specimens failed to support the evidence that CD19 + CD5 + B cells were a negative prognostic factor for patient survival
Hernandez-Prieto et al. [39]	50-gene signature (humoural immune genes)	Stage I or II, Adenocarcinoma, Squamous Cell Carcinoma, Adeno-squamous and Large Cell Carcinoma	Microarray	162	24	Whole genome microarray data from resected NSCLC specimens permitted molecular classification, whereby three clusters were identified, one of which had significantly higher recurrence free survival (RFS); the majority of the expressed genes were related to B cell immune response (B cell lineage genes such as Ig molecules, B cell receptor CD79a, CD19 lineage marker and B cell specific transcriptional co-activator POU2AF1 and marginal zone B1 cells) and these were over-expressed in the low risk sub-group. This gene signature and CD20 positivity were predictors of RFS; *p* < 0.0001 and *p* < 0.05, respectively
Schmidt et al. [43]	60-gene signature (humoural immune genes)	Stage I–IV, Adenocarcinoma, Squamous Cell Carcinoma	qRT-PCR Microarray	196	120	The 60-gene signature was significantly associated with longer survival of NSCLC, HR = 0.786, *p* < 0.001. This prognostic relevance was restricted to Adenocarcinoma, *p* = 0.002
Iglesia et al. [37]	60-gene signature (humoural immune genes)	Stage I to IV, Adenocarcinoma, Squamous Cell Carcinoma	mRNA- sequencing	504	ND	Expression of the 60-gene signature predicted improved overall survival in Adenocarcinoma, HR = 0.71, *p* = 0.00078
Mount et al. [38]	24-gene signature (humoural immune genes)	Stage I–III, Squamous Cell Carcinoma	Microarray	130	ND	B-cell related genes were predominantly found in early stage Squamous Cell Carcinomas. For stage I–II overall survival, *p* < 0.001
Liu et al. [39]	B-cell specific signatures	Adenocarcinoma and Squamous Cell Carcinoma (staging unavailable)	mRNA sequencing	980	ND	Deconvolution of bulk gene-expression data from a NSCLC cohort revealed that adenocarcinomas lacking memory B cell infiltration were associated with a poor prognosis at early clinical stage and, moreover, in squamous cell carcinomas the expression of T follicular helper (Tfh) cells was associated with a favourable prognosis
Faruki et al. [40]	B-cell specific signatures	Stage I–IV, Adenocarcinoma, Squamous Cell Carcinoma	mRNA sequencing or Microarray	933	ND	B cell infiltration was associated with a better prognosis in squamoid subtypes relative to the other subtypes but this was still not significant; HR = 0.747, *p* = 0.062.
Gentles et al. [41]	B-cell specific signatures	Stage I–IV, Adenocarcinoma, Squamous Cell Carcinoma, Large Cell Carcinoma, Small Cell Lung Carcinoma.	mRNA sequencing or Microarray	1336	185	B cell presence (memory, naive and plasma) denoted favourable outcomes for Adenocarcinoma, whereas memory and plasma cell presence denoted adverse outcome in Squamous Cell and Large Cell Carcinoma, respectively

MEDLINE Search 1998–2019 retrieved the papers populating Table 1

*IHC* immunohistochemistry, *IF* immunofluorescence, *qRT-PCR* quantitative real time polymerase chain reaction

In Table 1, the majority of studies have used a combination of immunofluorescence or immunohistochemistry to identify these cells in tissue. Of the 28 studies identified, 18 have used IHC as a primary method, mRNA sequencing has been employed in 4 studies and 2 have employed qt-PCR [37–41]. An anti-BCL-6 antibody and an anti-CD21 antibody were utilised to identify Germinal Centre B cells and mature B cells, respectively. Immunohistochemistry was used to determine B-cell density, location and phenotype within NSCLC tissue [8]. Studies have utilised PCR microarray and Mrna-sequencing techniques to identify primarily humoral immunity related gene signatures in NSCLC specimens [42, 43]. CD20 + B-cell infiltration has been shown to be positively prognostic in NSCLC by a number of different groups [38–43]. Disease-free and overall survival was significantly higher in non-smokers with non-squamous NSCLC [45]. Significantly improved survival has also been shown in large cell carcinomas with higher degrees of CD20 + B-cell infiltration [35]. Associations have been made between TLS (GCs forming as ectopic foci of follicular B cells and clusters of mature DC-Lamp^+ve^ Dendritic cells and T cells in cancer tissue, in response to antigen stimulation) in NSCLC and improved long-term survival. The presence of both types of antigen-presenting cells and mature dendritic cells in these TLS strongly predicts the outcome of patients [7, 44]. A low density of both follicular B cells and mature dendritic cells allows the identification of patients at high risk of poor survival. A higher prevalence of intra-tumoural GC formation was found in NSCLC stage I tumours compared with higher stage (II–IV) tumours (*p* < 0.02) [8]. In a recent study [49] the expression of a tumour-induced plasmablast-like B-cell signature (TIPB) was significantly correlated with the expression of CD8a signatures and the density of CD8 + cells. High expression of the TIPB signature was correlated with overall survival in the melanoma TCGA data set. Importantly, a cohort of melanoma patients treated with anti-CD20 antibodies, showed significant on-treatment down-regulation of the TIPB signature: the signature was highly correlated with tumour inflammatory score, interferon gamma and T cell effector signatures all of which significantly decreased on anti-CD20 therapy. There was a marked depletion in both CD4 + and CD8 + cell density at the invasive tumour-stroma margin and a reduction in the TLS area, an effect which was prolonged. In support of this data suggesting the importance of B cells in a successful anti-cancer immune response, long-term follow-up of CD20 depletion with Rituximab in patients with lymphoma, it was shown that CD20 depletion was an independent risk factor for the development of secondary solid tumour malignancy in both univariate and multivariate analyses [50].

Finally, the prognostic impact of follicular B cells was evaluated in two patient cohorts; early stage untreated NSCLC and advanced stage NSCLC treated with neoadjuvant chemotherapy. “Foll-B-Hi” patients had significantly prolonged survival in early stage disease, (97% DFS at 4 years compared with 62% in the “Foll-B-Lo” group), and in advanced stage disease, a benefit was demonstrated albeit not significant (56 month median DFS compared with 23 months in the “Foll-B-Lo” group). The global increase in follicular B-cell density was associated with an overall increase in mature DC density. When the combined immune populations were taken into account and correlated with survival, “Foll-B-Hi/mDC-Lamp^HI^” patients had the highest median survival, 100% of early stage patients (*p* < 0.04) and 55% of advanced disease patients (*p* = 0.007) were alive after a follow-up of 50 and 60 months, respectively [44]. “Foll-B-Lo/mDC-Lamp^LO^” patients had the worst prognosis.

Some studies have not demonstrated a prognostic impact of B-cell density on NSCLC outcomes [48, 51–54]. However, it may be that the lack of prognostic impact may relate to the high density of Bregs in such studies and it would appear to be essential that Breg density be considered separately [48, 51, 52]. An explicit analysis of whether Breg density is negatively prognostic for outcome, however, has not been performed. Finally, the prognostic impact may be dependant not only on enumeration of the appropriate B-cell subsets (TIMPs, follicular) but also by enumeration of tumour-associated B cells in the appropriate compartment, as opposed to analysis of un-segmented tumoural B-cell density. Most B cells are found at the invasive tumour-stroma margin, and it is here that the cancer cells are likely to polarise B cells to the immune-stimulatory TIPB phenotype.

## B-cell functionality: human NSCLC

### Antibody specificity and antigen presentation

Tumour antigen-specific B-cell responses are evidenced by the production of tumour-specific antibody and the oligo-clonality of TIL-Bs in the TLS [55]. B cells cultured from TLS’ have been shown to produce tumour-specific IgG and IgA antibodies [44]. LAGE-1 was identified as the most immunogenic tumour antigen in NSCLC, followed by MAGE family antigens, p53 and NY-ESO-1 [44]. Plasma cell and Ig expression associates favourably with outcome in NSCLC [43, 56, 57]. IgG4 + plasma cell infiltration correlates favourably with prognosis [57].

In NSCLC tissue TIL-Bs present antigen to CD4 + TILs with resultant effector responses [55]. CD4 + T cells, B cells and DCs were co-cultured together with protein ± co-stimulation with an anti-CD40/anti-CD28 antibody. Anti-HLA-DR, -DP and –DQ was used to block MHC class II antigen presentation. T cell responses were stratified according to “activated” (spontaneous presentation of antigen to CD4 + T cells), “antigen-associated” (presentation of antigen following re-stimulation by the antigen itself) and “non-responsive”. Activated TIL-Bs (CD19 + CD20 + CD69 + CD27 + CD21 +) and antigen-associated B cells mediated an effector T-cell response (IFN-γ producing CD4 + T cells). Conversely, exhausted phenotype TIL-Bs (CD19 + CD20 + CD69 + CD27T − CD21−) were associated with a regulatory T-cell phenotype (Foxp3 + CD4 + TILs) [55]. Exhausted TIL-Bs were still able to antigen present but controlled host damage from chronic antigen exposure by inducing a Treg phenotype and ultimately dampening anti-tumour immunity [55].

### Immunosuppression

In NSCLC, significantly higher frequencies of peripheral Bregs (CD19 + CD24^hi^ CD27 +) and CD19 + IL-10 + B cells were detected compared with healthy controls [58]. IL-10 + B-cell infiltration has positively correlated with CD25 + Treg expression and advanced clinical stage [59]. Multiple subsets exist with similarities in effector function and phenotype; in humans Br1 cells (CD19 + CD25^hi^ CD71^hi^) are strongly IL-10 positive. The differentiation of activated B cells into plasma cells is controlled by the expression of transcription factors, IRF4 and BLIMP-1 which are also critical for B-cell suppressive functions and some T cell suppressive functions (IRF4 expression in Tregs is dependent on Foxp3) [92]. B cells that co-express IRF4 and BLIMP-1 are the main source of B-cell derived IL-10 in vivo, and plasma cells are, therefore, a significant contributor, however, not all plasma cells produce IL-10 [60]. Currently, the signals that are required for differentiation into these regulatory B cells are not known.

Numerous studies have phenotypically characterised various Breg subsets in health and disease, this is displayed in Table 2 [58, 59, 61–80]. The findings from these studies of human Bregs in cancer underscores the ability of different Breg subsets to mediate immunosuppression in support of tumour growth through a variety of mechanisms; suppressive cytokine production (IL-10, TGF-β, IL-35), suppression of T cells and NK cells and the expansion of suppressive Tregs and myeloid-derived suppressor cells [30], expression of inhibitory ligands such as PD-L1 to dampen anti-tumour immunity [81] and STAT3 mediated promotion of angiogenesis and Treg augmentation [22, 68, 82]. It is unclear whether Bregs enhance tumour progression directly or if an increase in the Breg population is merely reflective of the immune response being mounted against the tumour [83].

**Table 2:** Human breg phenotypes in cancer and disease

Phenotype	Mechanism of suppression	Disease process	Author
CD19 + CD24hi CD38hi (Immature subtype)	IL-10, PD-L1	SLE	Blair et al. [61]
	CD1d	SLE	Bosma et al. [62]
	IL-10, TGF-P, CD40/CD40L	Hepatocellular carcinoma	Shao et al. [63]
	TGF-P	Breast cancer, Gastric cancer	Olkhanud et al. [64], Wang et al. [65]
	IL-10	Non-small cell lung cancer, Oesophageal cancer, Ovarian cancer	Liu et al. [59], Qian et al. [66], Zhou et al. [58], Wei et al. [67]
CD19 + CD5+	IL-10, STAT3	Lung, Prostate cancer	Zhang et al. [68]
	IL-10, TGF-P	Breast cancer	Lee-Chang et al. [69]
CD19 + CD5 + CD1dhi	IL-10	Chronic inflammation within gut-associated lymphoid tissue	Yanaba et al. [70], Mizoguchi et al. [71]
CD19 + CD35+	IL-35	Pancreatic Cancer	Wang et al. [72], Pylayeva-Gupta et al. [73]
CD19 + CD24hi CD27 + (B10 cells)	IL-10	SLE, Rheumatoid arthritis, primary Sjogren’s Syndrome, Multiple sclerosis, Autoimmune vesiculobullous skin disease	Iwata et al. [74]
CD19 + CD38 + CD1d + IgM + CD147 + (GrB + B cells)	IL-10, GranzymeB	Breast, Ovarian, Cervical, Colorectal, Prostatic carcinomas	Lindner et al. [75]
CD19 + CD25hi CD71hi CD73lo (Br1 subtype)	IL-10, IgG4	Human model of allergic inflammation in response to bee venom antigen	van de Veen et al. [76]
CD19 + CD24hi CD27int CD38hi (Plasmablast subtype)	IL-10	Healthy human donor peripheral blood	Matsumoto et al. [77]
CD39 + CD73+	Adenosine	Healthy human donor peripheral blood	Saze et al. [78]
(iBreg subtype)	TGF-P, IDO	Healthy human donor peripheral blood	Nouel et al. [80]
CD19 + TIM-1+	IL-10	HIV-1 Infection	Liu et al. [79]

MEDLINE Search 2000–2019 retrieved the papers populating Table 2

## B-cell milieu in NSCLC

B cells can exist in a continuum of naïve cells to terminally differentiated plasma cells within the TME and more specifically within the TLS [44]. Determining the ratio between these so-called “anti-tumour” TLS derived TIL-Bs and the “pro-tumour”, inhibitory Bregs is important to understand the biology and long-term outcome from this disease. This balance is likely influenced by the microenvironmental cues which play a role in determining B-cell polarity. CXCL13 and Lymphotoxin have been identified as two factors critical to the formation and development of lymphoid follicles in the gut [84], and in lung cancer, B cells produce CXCL13 and Lymphotoxin via TLR4 signalling which acts as a positive feedback loop to support the formation and high density of TLS [85, 86]. CXCR5 expressing B cells stimulated by CXC13 coupled CpG-ODN can trigger the cytolytic effect of CD8 + T cells leading to the abrogation of metastasis in 4T1.2 tumour-bearing mice [23]. Resveratrol, Lipoxin, Glucosides of Paeony have also inhibited Bregs through STAT3 and/or ERK inactivation leading to a reduction in IL-10 and TGF-β levels thus exerting an anti-tumour effect [87]. B-cell homeostasis and thus polarity will largely be determined by the degree of inflammation within the tumour, factors such as tissue hypoxia, intra-tumoural vascularity, cytokine milieu and cellular infiltration are all factors which are likely to exert control over the pro versus anti-tumour B-cell balance but as yet there is little evidence describing the Breg/B effector ratio in tumour biology, and this is likely due to the transient inducible nature of Bregs.

## The interplay of B cells and checkpoint blockade

Immune checkpoint blockade antibodies have improved cancer therapy by overcoming the inhibition of T cell effector functions, yet a significant proportion of patients still do not respond to such therapies. In the first study to investigate whether B-cell density impacts outcome with checkpoint blockade, B-cell content was determined in 34 melanoma patients undergoing PD-1 blockade monotherapy and evaluated for response [88]. Dichotomising the patients at the median of CD20 positive cells in at least one histospot there was no difference in response or survival between those with high and low B-cell density. The comments made earlier about enumeration of specific B-cell populations in specific microenvironmental segments made earlier also apply here. B-cell depletion in the MC38 (colon carcinoma) and YUMMER1.7 (melanoma) models did not impact the efficacy of anti-PD-1 treatment. Anti-PD-1 outcomes were similar in *mu*MT mice (mice lacking B cells) and WT mice bearing MC38 tumours [88]. Larger data sets in other cancers need to be interrogated to fully understand whether there is any impact of specific intra-tumoural B-cell populations on the outcome of checkpoint blockade. This is particularly the case given earlier data showing that clinical benefit with ipilimumab was greater in melanoma patients with sero-positivity against NY-ESO [89]: as mentioned above, the CTags appear to be potent immunogens stimulating antibody responses. Furthermore, gene-expression profiling in urothelial carcinoma and melanoma patients undergoing both anti-PD-1/PD-L1 and anti-CTLA4 therapy identified a memory B-cell (MBL) signature which was significantly and reproducibly elevated in patients showing clinical benefit [90]. It significantly outperformed other immune cell signatures and remained significantly associated with outcome when including tumour mutational burden, copy number aberration burden and checkpoint expression. Samples enriched for an innate PD-1 resistance scores had significantly lower levels of MBL scores. The MBL score positively correlated with BCR heavy chain expression and the expression of T cell activation genes, MHC class II and genes responsible for B-cell proliferation and activation within the TME. Finally, high expression of the TIPB signature was associated with improved survival in melanoma patients treated with anti-PD-1, and plasmablast-like and naïve B-cell frequencies were significantly higher in patients responding to immune checkpoint blockade [49]. The use of anti-PD-1 treatment in murine models has shown to increase total IgG and OVA-specific IgG production in OVA-immunised mice [91]. The enhanced humoural response in these mice is thought to be mediated by CD4 + ICOS + T cells which are presumably of the T follicular helper (Tfh) phenotype that go on to augment terminal B-cell differentiation in the germinal centre [91]. PD-1/PD-L1 interactions between Tfh and Bregs control this axis [92, 93] and by blocking this checkpoint, Tfh cells are released from Breg-induced suppression. This demonstrates the importance of heterogeneity of the B-cell repertoire and how checkpoint blockade can impact downstream immune responses by targeting select populations. Importantly, none of the above studies have examined B-cell density as predictive of response to checkpoint blockade in NSCLC or to combination chemo/immunotherapy which has become a 1st line standard of care in this disease.

Given that PD-1 is expressed on B cells and can limit B-cell responsiveness [94, 95], and furthermore, the certain autoimmune conditions are mediated via auto-antibody formation, the association of B-cell sub-populations with checkpoint blockade toxicity has become a focus of investigation. Das et al. demonstrated in melanoma patients, a detectable decline in circulating B-cell numbers together with an increase in CD21^LO^ B cells and plasmablasts after the first cycle of combination checkpoint blockade therapy [96]. These treatment induced changes in B-cell numbers preceded and correlated with both the frequency and timing of immune related adverse events (irAE). Early B-cell changes correlated with a higher rate of grade 3 or higher irAEs 6 months after starting treatment. Contrastingly, several groups have shown through case report series’ that B-cell depletion therapy using Rituximab successfully treated B-cell-mediated irAEs in NSCLC [97, 98], SCLC [99], Melanoma [100] and Urothelial Carinoma [101]. This recapitulates the idea of B-cell enumeration and selective targeting of certain microenvironmental B-cell populations. Bregs which by nature are immunosuppressive and dampen down inflammation would limit anti-tumour activity in response to PD-1 blockade but effector B-cell populations with robust humoural responses and T cell activation mechanisms are driving autoimmunity and irAE in response to PD-1 blockade. These intriguing analyses need to be validated, extended and applied to other cancers amenable to checkpoint blockade and the mechanisms underlying these observations identified.

## Conclusions

Studies in mouse models of pre-malignancy suggest that B-cell-mediated inflammation may be important in promoting the progression to invasive malignancy. Given the huge promise of reversing the pre-malignant phenotype to reduce the cancer burden, there is an urgent need to understand the role of B cells in human metaplasia, dysplasia and in situ cancer and how they mediate progression through these stages to decide whether B-cell-directed strategies may be of value in reducing the progression of pre-malignancy.

Studies examining B cells with a regulatory phenotype (Bregs) consistently suggest that Breg infiltration may enhance tumour progression. The factors that induce Bregs in human malignancy need to be defined. Specifically are there particular microbes, TLR ligands or cancer cell produced cytokines in the TME that polarise B cells to a Breg phenotype [14, 102]. Currently used B-cell depleting antibodies cannot distinguish between effector and regulatory B-cell subsets; therefore, meticulous phenotypic characterisation and study of this subset in the TME [14, 102] is required to identify Breg specific targets that can be exploited to selectively deplete Breg populations but more fundamentally to fully understand the role of Bregs in human cancer. There are some current potential anti-Breg strategies. In vivo murine studies have displayed selective Breg depletion using LXA4 without affecting conventional B-cell proliferation, differentiation and germinal centre formation thus promoting anti-tumour responses [87]. An alternate to Breg depletion would be repolarisation of this subpopulation into B effector cells, as has been shown with TLR9 ligands in vitro [22, 23]. Adoptive transfer of CpG-pulsed B cells with effector phenotypes into patients with established cancer could be employed to shift the balance in favour of an anti-tumour B-cell response within the TME.

More work is needed to understand the anti-tumour impact of antibodies against tumour associate antigens, particularly CTags which appear to be strong immunogens, and to identify new humoural immunity targets. The disappointing results of the MAGRIT trial vaccinating NSCLC patients in the adjuvant setting [103] should not be taken as suggesting that harnessing the anti-tumour antibody response should be deprioritised: mono-epitopic vaccination as cancer therapy has a long history of failure. Multi-valent vaccines, preferably against personalised B-cell antigens, are one option. Building on the model of the chimaeric antigen receptor T cells (CART), highly specific B-cell receptors to critical tumour antigens could be cloned into autologous B cells and transferred into patients with resultant high specificity and high affinity anti-tumour Ig production. Alternatively, antibodies could be produced ex vivo and adoptively transferred. Given the role of B-cell PD-1 expression in mediating B-cell hypo-responsiveness, the role of PD-1 blockade in augmenting these strategies should be explored, as a research priority. Understanding B-cell biology will help to refine the understanding behind the effects of checkpoint blockade on the immune milieu. Toxicity from these therapies is the Achilles heel of this treatment strategy. As was alluded to earlier, work in mice and humans has demonstrated that PD-L1^hi^ Bregs play a role in the suppression of humoral immunity through Tfh cell regulation; moreover, these cells are resistant to classical anti-CD20 therapy [93]. Firmly understanding the ontogeny of these B cells and their relationship to other B-cell subsets, including other Breg phenotypes is of paramount importance if we hope to be able to refine therapeutic strategies so as to augment anti-tumour protective immunity and dampen down autoimmune and hence toxic responses.

Finally, large scale prospective and careful B-cell sub-type specific and microenvironment segment specific analyses are required in lung cancer and in other cancers to clarify the role of B cells in modulating the responsiveness to checkpoint blockade and in mediating the toxicity to these therapies. These studies will define the role of B-cell-targeted strategies in augmenting the activity of, reducing resistance to and the ameliorating toxicity of this crucial class of anti-cancer agents.

## Data Availability

All data and materials used in constructing this manuscript can be found through a Medline search using Pubmed, all data is referenced in the section below.
